# *Echinococcus granulosus* infection reduces airway inflammation of mice likely through enhancing IL-10 and down-regulation of IL-5 and IL-17A

**DOI:** 10.1186/s13071-014-0522-6

**Published:** 2014-11-20

**Authors:** Hui Wang, Jun Li, Hongwei Pu, Bilal Hasan, Jinfeng Ma, Malcolm K Jones, Kan Zheng, Xue Zhang, Haimei Ma, Donald P McManus, Renyong Lin, Hao Wen, Wenbao Zhang

**Affiliations:** State Key Laboratory Incubation Base of Xinjiang Major Diseases Research, Clinical Medical Research Institute, The First Affiliated Hospital of Xinjiang Medical University, Urumqi, Xinjiang 830054 China; Department of Immunology, Xinjiang Medical University, Urumqi, 830011 Xinjiang China; Laboratory of Respiratory Physiology and Pathology, Traditional Chinese Medicine Hospital affiliated with Xinjiang Medical University, Urumqi, Xinjiang 830054 China; Department of Epidemiology and Health statistics, School of Public Health Xinjiang Medical University, Urumqi, Xinjiang 830054 China; School of Veterinary Sciences, The University of Queensland, Queensland, Warrego Highway, Gatton, Qld 4343 Australia; Molecular Parasitology Laboratory, Infectious Diseases Division, QIMR Berghofer Medical Research Institute, Brisbane, Queensland Australia

**Keywords:** Allergic asthma, *Echinococcus granulosus*, Cystic echinococcosis, IL-5, IL-10, IL-17A, Airway inflammation

## Abstract

**Background:**

Cystic echinococcosis (CE) is a near cosmopolitan zoonosis caused by the larval stage of the dog tapeworm *Echinococcus granulosus. E. granulosus* infection induces a polarized T-helper type 2 (Th2) systematic immune response in its intermediate hosts. However, it is not known whether the infection modulates lung inflammation by regulating local immune response. In this study, we examined the effects of *E. granulosus* infection on mouse ovalbumin (OVA)-induced asthma model.

**Methods:**

BALB/c mice were intraperitoneally transplanted with 50 small *E. granulosus* cysts cultured *in vitro*. At 3 months post-inoculation, the mice were sensitized and challenged with ovalbumin (OVA). For histopathological studies, hematoxylin eosin and periodic acid schiff staining was used to examine the inflammatory cells infiltration and goblet cells hyperplasia, respectively. Cytokine levels were measured by mouse cytometric bead array (CBA) Kit and quantitative RT-PCR and other molecular biological approaches. Airway hyperresponsiveness was assessed in response to increasing doses of methacholine. Serum immunoglobulins were determined by ELISA.

**Results:**

*E. granulosus* infection significantly increased Th2 and Treg cytokine levels in serum and lung tissues, but down-regulated the expression of IL-5 in the lungs and IL-17A in serum and lung tissues of asthmatic mice sensitized and challenged with OVA. Histological staining of lung tissues showed that *E. granulosus* infection significantly reduced the severity of OVA-induced airway inflammation including reduction of eosinophil cell infiltration and mucus production. The *E. granulosus* infection also reduced eosinophil accumulation induced by OVA in bronchoalveolar lavage fluid (BALF) and also ameliorated airway hyperresponsiveness, a hallmark symptom of asthma.

**Conclusions:**

*E. granulosus* infection remarkably reduces the severity of OVA-induced airway inflammation likely through enhancing IL-10 and down-regulation of IL-5 and IL-17A.

**Electronic supplementary material:**

The online version of this article (doi:10.1186/s13071-014-0522-6) contains supplementary material, which is available to authorized users.

## Background

Cystic echinococcosis (CE) is a near cosmopolitan zoonosis caused by the larval stage of the dog tapeworm *Echinococcus granulosus*. This disease is highly endemic and is a major public health problem in Central Asia, the countries around the Mediterranean, Northern Africa and South America [1,2]. The transmission depends on social and environmental factors [3]. Sero-epidemiological surveys in the hyper-endemic areas of highland Peru and western China, have revealed 10-30% seropositivity in villagers [4-6], indicating that large numbers of individuals are exposed to *E. granulosus* infection in these communities, even though only 1-9% of the population have cysts detected by ultrasonography [7,8]. One significant feature of CE is the fact that the larval cysts of *E. granulosus* are able to survive in intermediate hosts for a very long time (up to 53 years in humans) without apparently causing pathological damage in host tissues surrounding the cyst [9,10], indicating that the parasite can modulate the host immune response towards a chronic state. In fact, it has been shown that *E. granulosus* cysts induce an early (in the first two weeks) Th1-type cytokine profile (IFN-gamma and IL-2), followed by a shift toward a Th2-type profile (IL-4, IL-5, IL-6, IL-10 and IL-13) in a mouse model [10-12]. CE patients normally show a predominant Th2 profile and also found an elevated serum IgE [13]. Normally a Th2 response and IgE are associated with an increase in asthmatic responses [14,15]; therefore, an *E. granulosus* infection is likely to boost the airway allergic response. However, there are no reports that show that inhabitants living in *E. granulosus* -endemic areas are at increased risk of allergic disease. Whereas schistosomiasis, caused by trematode blood flukes, is characteristically associated with a predominant Th2 cytokine production combining eosinophilic and IgE responses [16], schistosome infections ameliorate atopic disorders in humans [17,18]. Furthermore, epidemiological studies have shown that inhabitants in schistosomiasis-endemic areas had less incidence of asthma, compared with those living in non-endemic regions [19]. This phenomenon was first demonstrated in mouse models of *Schistosoma mansoni* [20] and the nematode, *Trichinella spiralis* [21], which showed that these infections protected mice from OVA-induced airway reactivity.

In this study, we used our established secondary CE infection mouse model [22] to determine whether *E. granulosus* infection can impact on allergic asthma inflammatory responses induced by ovalbumin (OVA). We showed that the infection significantly suppressed OVA-induced eosinophilic airway inflammation through enhancing the level of IL-10 and down-regulation of IL-17A. As far as we are aware, this is the first report of a study on *E. granulosus* infection impacting on allergic asthma inflammatory responses.

## Methods

### Experimental animals

Pathogen-free female BALB/c mice, aged 6–8 weeks (about 20 g in weight), were purchased from Beijing Vital River Laboratory Animal Technology Company Limited, and raised in the animal facility of the First Affiliated Hospital of Xinjiang Medical University (FAH-XMU). All experimental protocols involving mice were approved by the Ethical Committee of FAH-XMU (Approval No IACUC-20120625003).

### Animal infection and murine models of allergic asthma

All BALB/c mice were randomly divided into four groups with 10 mice in each group comprising: (1) negative control group administrated with PBS only (PBS); (2) *E. granulosus* infection group (Eg); (3) ovalbumin (OVA) sensitization and challenge group (OVA); (4) *E. granulosus* infection plus OVA sensitization and challenge group (Eg + OVA). To obtain mice successfully infected with hydatid cysts, we pre-cultured *E. granulosus* protoscoleces *in vitro*, which generated small cysts (200–300 μm in diameter), as previously described [22]. The mice in Eg and Eg + OVA groups were each intraperitoneally (i.p) transplanted with 50 small hydatid cysts, suspended in 0.4 mL of RPMI 1640 medium, using a 1.0 mL syringe assembled with a 20 gauge needle. After 90 d of cyst transplantation, two groups (Eg + OVA and OVA) of mice were each sensitized by intraperitoneal (i.p.) injections with 100 μg of OVA (Sigma–Aldrich, St. Louis, MO, USA) on days 91 and 104 post-cyst-transplantation mixed with 1 mg of aluminum hydroxide (Sigma–Aldrich) in 200 μL of PBS. These two groups of mice were then challenged with 2% (w/v) OVA in PBS via the airway and the oral route for 30 min using a nebulizer each day for 3 d from day 111 to day 113. Negative control and *E. granulosus* infection group mice were sensitized and challenged with PBS only.

### Measuring airway hyperresponsiveness (AHR) to methacholine

The day after the final OVA challenge, the mice were analyzed using non-invasive lung function measurements (BUXCO WBP, USA) to assess AHR. The pulmonary assessment of enhanced pause (Penh) value was assessed by barometric whole body plethysmography in response to increasing doses of aerosolized methacholine (Mch) (acetyl β-methylcholine chloride; Sigma-Aldrich) challenge. Briefly, the mice were permitted to acclimate for 5 min, PBS aerosol was administered to establish baseline readings over 3 min, and then mice were subsequently treated with a series of increasing concentrations (0, 3.125, 6.25, 12.5, 25, 50 mg/mL) of Mch for 2 min each dose to induce bronchoconstriction. After each nebulization, recordings were obtained for 3 min, and the Penh values measured during each 3 min period were averaged. Results were expressed as the percentage increase in Penh following challenge with each increasing concentration of Mch. Twenty-four hours later, mice were euthanized with carbon dioxide and samples including lung tissues, spleen, BALF and blood (see the following sections for details) were taken for further immunological analysis.

### Bronchoalveolar lavage fluid (BALF)

BALF was collected by cannulating the trachea and lavaging the mouse lungs with 1.5 mL of cold PBS. The recovered BALF samples were centrifuged at 1500 rpm for 5 min at 4°C and then the cell pellets were resuspended in 800 μL of PBS and centrifuged for 5 min at 1500 rpm at 4°C. The cells were resuspended with 100 μL of PBS for counting numbers of eosinophils, neutrophils, lymphocytes, and macrophages using a Blood Corpuscle Analyzer (Mindray BC-5300Vet, ShenZhen, China).

### Histological examination

For histopathological studies, the lung tissues of the mice were fixed in 4% (v/v) paraformaldehyde in PBS and paraffin-embedded for sectioning. Serial sections of 5 μm thickness were cut and stained with hematoxylin and eosin (H&E) to examine inflammatory cell infiltration and changes in general histology. The sections were also stained with Periodic Acid Schiff reagent (PAS) (BASO Diagnostic, Zhuhai, China) to examine goblet cell hyperplasia in the bronchial epithelium. The severity of inflammatory cell infiltration was determined by counting blindly the number of peribronchial eosinophils using the 5-point scoring system described by Myou *et al.* [23], as follows: 0- no eosinophils infiltrated; 1- mild with few eosinophils infiltrated; 2- medium with discontinuous single layer of eosinophils attached to the peribronchial zones; 3- moderate infiltrate, with one layer of eosinophils around the peribronchial zones and 4- marked infiltrate, with more than one layer of eosinophils and a changed morphology of peribronchial zones.

To determine the extent of mucus production, goblet cell hyperplasia in the airway epithelium was quantified blindly using the 5-point grading system described by Tanaka *et al*. [24]. The adopted grading system was: 0- no goblet cells; 1- <25% goblet cells in cell population; 2- 25–50%; 3- 50–75%; 4- > 75% of goblet cells. Scoring of inflammatory cells and goblet cells was performed in at least three different fields for each lung section. All the pathology sections were subjected to blind examination by two pathologists.

### Cell preparation

Mice were sacrificed 24 h after Mch challenge, Peritoneal exudate cells (PECs) were harvested by thorough washing of the peritoneal cavity with 5–6 mL of PBS for three times. The recovered PECs were centrifuged at 1500 rpm for 5 min at 4°C and the cell pellets were resuspended in 100 μL of PBS in order to count eosinophils, neutrophils, lymphocytes, and macrophages using the same protocol and instrument as described in BALF section. To isolate splenocytes, spleens were squeezed using a plunger of a 5 mL syringe on a 70 μm cell strainer (BD Biosciences, CA, USA) placed in a Petri-dish containing 1 ~ 2 mL RPMI 1640. The pass-through tissues were suspended with 10 mL of PBS and transferred into a 15 mL centrifuge tube. After centrifugation, the cell pellet was resuspended and hemolyzed with 10 mL of 1 × RBC lysis buffer. The cells were resuspended in RPMI 1640 after a further wash with PBS.

### Flow cytometry

The splenocytes were resuspended in FBS buffer (BD Pharmingen, San José, CA, USA). For staining and counting CD4^+^CD25^+^FoxP3^+^ cells, spleen cells were treated using the provided protocol of the Mouse Regulatory T Cell Staining Kit (BD Biosciences). For CD4^+^IL-17A^+^ staining, a concentration of 1 × 10^6^ cells/mL in media were stimulated with PMA/Ionomycin (at 50 ng/mL and 1 μg/mL respectively) in the presence of BD GolgiStop™ Protein Transport Inhibitor for 5 h. Then the cells were collected and stained using anti-mouse antibodies, FITC-CD4 and PE-IL-17A (BD Biosciences). The cells were protected from light throughout the staining procedure and storage. The fluorochrome intensity was measured by a FACS Aria II flow cytometer (BD Biosciences) and analyzed using CellQuest software (BD Biosciences).

### Measurement of cytokines in the culture of spleen cells and serum

Single-cell suspensions of splenocytes (1 × 10^7^cells/mL/well) were plated in 24-well tissue culture plates in RPMI 1640 (Gibico) supplemented with 10% (v/v) heat-inactivated FBS (Gibico), 100 U/mL penicillin, and 100 μg/mL streptomycin (Hyclone), and cultured alone or with Con A (5 μg/mL; Sigma-Aldrich) at 37°C in an atmosphere of 5% CO_2_ in air for 3 d. The culture supernatants were collected, centrifuged at 2500 × g for 5 min at 4°C, and cytokine levels were measured by mouse cytometric bead array (CBA) Kit (BD Biosciences). Briefly, 50 μL of samples (splenocyte culture and serum) or known concentrations of standard samples (0–5000 pg/mL) were added to a mixture of 50 μL each of capture antibody bead reagent and phycoerythrin (PE)-conjugated detection antibody. The mixture was then incubated for 2 h at room temperature in the dark and then washed to remove unbound detection antibody. Data were acquired using a FACS AriaIIflow cytometer and analyzed using CBA software 1.1 (BD Biosciences).

### Quantitative RT-PCR

Total RNA was isolated from the lung tissues of mice using Trizol reagent. After removing contaminated DNA from the isolated RNA using DNaseI (Fermentas, Vilnius, Lithuania), 1 μg of total RNA was reverse transcribed into cDNA in 20 μL reaction mixtures containing 200 U of moloneymurine leukemia virus reverse transcriptase (MMLV, Promega, Madison, USA); 100 ng of oligo (dT) primers; and 0.5 mM each of dNTPs. The resulting templates were quantified by real-time PCR (iQ5, Bio-Rad, Hercules, CA) using SYBR Green PCR Kit (Qiagen, Cat. No 204054) in conjunction with mouse gene-specific primers for IL-2, IL-4, IL-5, IL-17A, IL-10, IFN-γ and GAPDH (Additional file 1: Table S1).

For each sample, both the housekeeping and the target genes were amplified in triplicates using the following cycle scheme: initial denaturation of the samples at 95°C for 15 min; 40 cycles at 95°C for 15 s,60°C for 30 s and 72°C for 30 s. Fluorescence was measured during every cycle, and a melting curve was used to analyze the specificity and purity of PCR products amplified by increasing the temperature from 55 to 95°C (in 0.5°C increments). A defined single peak was used to confirm the specificity of the amplification. We used the serial dilution PCR products of IL-2, IL-4, IL-5, IL-17A, IL-10, IFN-γ and GAPDH for making their respective standard curve. The relative gene expression values were normalized to the expression values of GAPDH within each of the samples.

### Western blot analysis

Fifty mg of lung tissue from each mouse was suspended in RIPA buffer (Bio Teke Corporation, Beijing) for 30 min on ice to isolate proteins for Western blot analysis. The lung tissue was then homogenized using a Polytron (PRO Scientific Lnc, Oxford). After centrifugation, each supernatant obtained from the tissue extract containing 50 μg of total proteins was separated using 12% (w/v) SDS-polyacrylamide gel electrophoresis (SDS-PAGE) and then electrophoretically transferred to a PVDF membrane (Amersham Biosciences, Buckinghamshire, UK). The membranes were incubated for 60 min at room temperature with the appropriate primary antibodies: anti-eosinophil major basic protein (EMBP) (dilution 1:200, Santa Cruz Biotechnology, Santa Cruz, CA), anti-IL-5 (dilution 1:800) (Bioworld Technology, St. Louis Park, MN, USA), IFN-γ (dilution 1:200, proteintech, Chicago, USA), and anti-GAPDH (dilution 1:1000, Cell Signaling technology, MA, USA). Immunoreactivity was detected using the WesternBreeze Kit (Invitrogen, CA, USA). The expression levels of respective proteins (in “relative units”) in the lungs of the PBS control group, *E. granulosus* infection group, Eg + OVA group and OVA group mice were quantified using Quantity One software (Bio-Rad) after scanning of the Western blot membranes. In the method, the band intensities reflected the average intensity of target protein over the area of the band normalized with that of GAPDH.

### Determination of serum immunoglobulin by ELISA

A standard microwell enzyme-linked immunosorbent assay (ELISA) was performed [25] to determine specific immunoglobulin antibody levels (IgG, IgG1, IgG2a, IgG2b, IgE) against hydatid cyst fluid (HCF) antigens and OVA-specific immunoglobulins (IgG1, IgG2a, IgE) in serum. HCF was collected from sheep hydatid cysts and concentrated 10 times using a vacuum dryer. ELISA plates (Costar, NY) were coated with HCF(10 μg/mL)or OVA(1 μg/mL), respectively. The sera were diluted with 1% (w/v) BSA to 1:200 for the detection of IgG and 1: 50 for IgE; the plates were incubated at 37°C for either 1 h (IgG) or overnight (IgE) at 4°C. The plates were then washed four times with PBS containing 0.1% Tween 20 (PBST), followed by adding second antibody conjugated with horse radish peroxidase (HRP) including goat anti-mouse IgG or IgG subclass (IgG1, IgG2a, gG2b) at 1:3000 and, goat anti-mouse IgE at 1:2500. The plates were then incubated for 1 h at 37°C, except for the detection of IgE levels where the plates were incubated overnight at 4°C. The plates were washed five times with PBST. The assays were developed in 2, 2-azino-di-(ethyl-benzithiozolinsulpho-nate) (ABTS) (Sigma) substrate solution for 30 min. The optical density (OD) of the color that developed in the plate was read at 405 nm using an ELISA reader (Thermo, MA, USA USA).

### Statistical analysis

Data were analyzed using the GraphPad Prism 5 software (Graph-Pad Software, Inc., USA). All values are presented as the means ± S.E.M (standard error of the mean). Statistical differences between groups were determined by One-way ANOVA followed by a Tukey’s test. Statistic significances were accepted when *p* <0.05.

## Results

### Infection model of *E. granulosus*

To obtain an even number of hydatid cysts in mice, we pre-cultured protoscoleces of *E. granulosus* into small hydatid cysts with an average diameter of 250 μm. We then intraperitoneally transplanted 50 of the small cysts into each of BALB/c mice. All the mice necropsied at 115 d post-inoculation were successfully infected with hydatid cysts, with a mean of 34.9 (20–49) cysts per mouse. The cysts were only existed in the peritoneal cavity of the mice. The procedure thus generated about 70% of cyst recovery with 100% of mice infected, showing a stable mouse infection model had been established. The mean diameter of the cysts was 4.4 ± 1.8 mm with the largest cyst being 18 mm in diameter.

### Effect of *E. granulosus* infection against OVA-induced eosinophil recruitment

**Figure 1:**
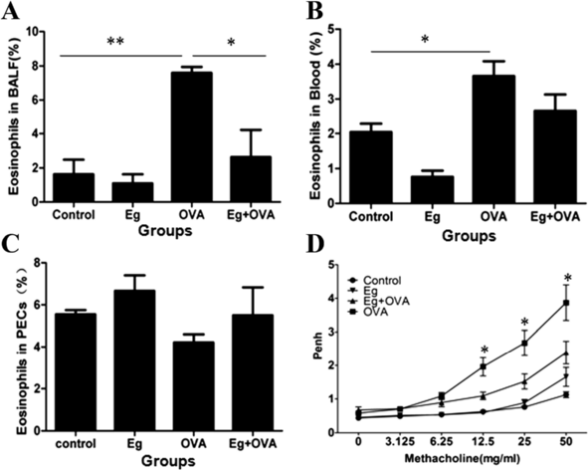
**Effect of** ***E. granulosus*** **infection on OVA-induced eosinophil recruitment and airway hyperresponsiveness.** Samples collected from the BALF **(A)**, blood **(B)** and PECs **(C)** of mice sensitized and challenged 24 h after the last OVA challenge(n = 5). The enhanced pause (Penh) was evaluated at baseline and after treatment with increasing doses of aerosolized methacholine (0–50 mg/ml). *, significant difference from OVA group, *p* <0.05 **(D)**. OVA, ovalbumin; BALF, bronchoalveolar lavage fluid; PECs, peritoneal exudate cells; control, PBS control group; Eg, *E. granulosus* infection group; Eg + OVA, *E. granulosu* infection plus OVA-induced asthmatic group; OVA ,OVA-induced asthmatic group. *p <0.05; **p <0.01.

To determine whether *E. granulosus* infection impacts on asthma progression given that the infection significantly regulates the host immune response [11,12], the mice infected with *E. granulosus* cysts were sensitized and challenged with OVA. The numbers of eosinophils in BALF, PECs and blood of the mice were counted 24 h after the last OVA aerosol challenge. Figure 1A showed there was a significant increase of eosinophils in the BALF of OVA challenged mice compared with that of the PBS controls; Eg + OVA mice did not have significantly increased numbers of eosinophils compared with the OVA challenged mice. In contrast, the numbers of neutrophils, macrophages, and lymphocytes were not significant difference between all groups of mice (data not shown). Figure 1B shows that blood eosinophil counts were also significantly increased in OVA challenged mice but not in PECs (Figure 1C). In addition, macrophages counts were significantly increased in the PECs in *E. granulosus*-infected mice compared with those in PBS control mice, indicating the lesions or the cysts may attract macrophages (data not shown).

### Effect of *E. granulosus* infection on airway hyperresponsiveness (AHR)

To evaluate the inhibitory effects of *E. granulosus* infection on AHR, the Penh was assessed by barometric whole body plethysmography in response to increasing doses of aerosolized Mch challenge. OVA-challenged mice showed a dose-dependent increase in the Penh values compared with the control mice after Mch inhalation (from 0 mg/mL to 50 mg/mL). However, the Penh values were lower in the mice infected with *E. granulosus* and OVA sensitized and challenged compared with the values of OVA mice. *E. granulosus* infected mice without exposure to OVA had no spontaneous AHR (Figure 1D).

### Histopathological analysis of the effect of *E. granulosus* infection on OVA-induced eosinophil infiltration and mucus production

**Figure 2:**
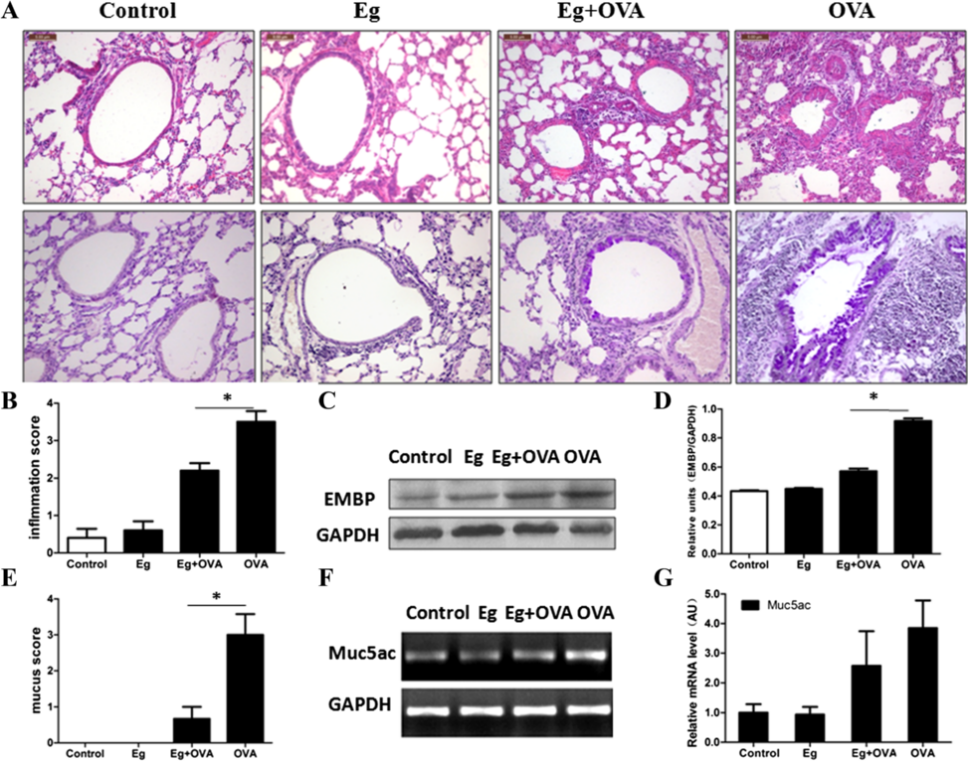
**Effect of** ***E. granulosus*** **infection on OVA-induced airway inflammation and mucus production.** The histological appearance of lungs after sensitization and challenge with PBS or OVA (magnification: 200×) **(A)**. Quantitative analyses of inflammatory cell infiltration and mucus production in the lung **(B,E)**. Western blotting showing relative expression of EMBP in different treatment groups **(C,D)**. Semi-quantitative analysis of mRNA transcripts for expression of Muc5ac **(F,G)**. The bars indicate mean ± S.E.M. for values obtained by densitometry analysis. *p <0.05,n =8 mice/group, in two separate experiments.

To further analyze the effects of *E. granulosus* infection on the histopathology of airway allergic inflammation responses induced by OVA, the lung tissues of the mice were histologically examined 24 h after challenge with Mch. The analysis showed that *E. granulosus* infection did not induce obvious changes in histology and pathology compared with the control mice. By contrast, OVA induced a marked infiltration of inflammatory cells, mainly eosinophils, into the peribronchiolar and perivascular regions of the lungs. For the Eg + OVA mouse group, only minimal cellular infiltration was noted around the bronchioles, with a smaller average infiltration of inflammatory cells (Figure 2A, B). PAS staining was performed on lung tissues to analyze the effects of *E. granulosus* infection on the hyperplasia of goblet cells in the peribronchial epithelia and mucus production in the lungs of mice given OVA. OVA-challenged mice developed marked PAS-positive goblet cell hyperplasia and mucus hypersecretion within the bronchi in the lung (purple colour) compared with the PBS control mice. Infection with *E. granulosus* substantially decreased the number of PAS-positive goblet cells in the peribronchial epithelia (Figure 2A, E).

We also examined the effect of *E. granulosus* infection on eosinophil major basic protein (EMBP), a constituent of eosinophil secondary granules, that is elevated in biological fluids taken from patients with asthma and other eosinophil-associated diseases [26]. *E. granulosus* infection reduced the increased EMBP expression in lung tissues (Figure 2C, D)) of OVA mice. Airway mucus hypersecretion originates from hyperplastic goblet cells that typically express Muc5ac [27]. However, statistical analysis showed that the expression level of Muc5ac mRNA (Figure 2F, G) between groups was the same.

### Effect of *E. granulosus* infection on cytokines in serum and culture of splenocytes

**Figure 3:**
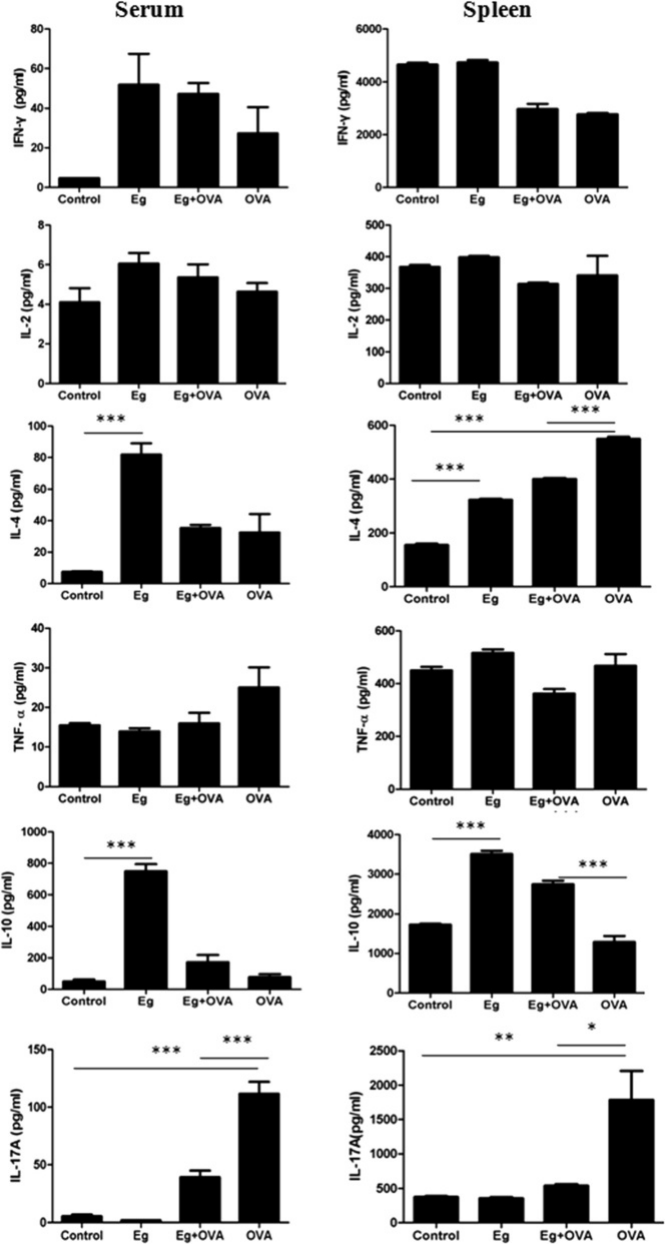
**Effects of** ***E. granulosus*** **infection on cytokine levels in serum and splenocytes cultured** ***in vitro*** **.** *, *p* <0.05; **, *p* <0.01; ***, *p* <0.001.

To determine whether *E. granulosus* infection impacts on the immune profile of systemic immunity, we measured the concentration of cytokines in the serum and the supernatants of splenocytes cultured *in vitro*. The infection induced a significant increase of IL-4 and IL-10 in serum and the culture of splenocytes, whereas, OVA induced a significant increase of IL-17A in serum and splenocyte supernatants compared with in the PBS control mice (Figure 3). However, compared to OVA induced group, the concentration of IL-17A in Eg + OVA group was down-regulated as shown by its reduced level in the serum and splenocyte cultures (Figure 3).

### Effect of *E. granulosus* infection on cytokines in the lungs

**Figure 4:**
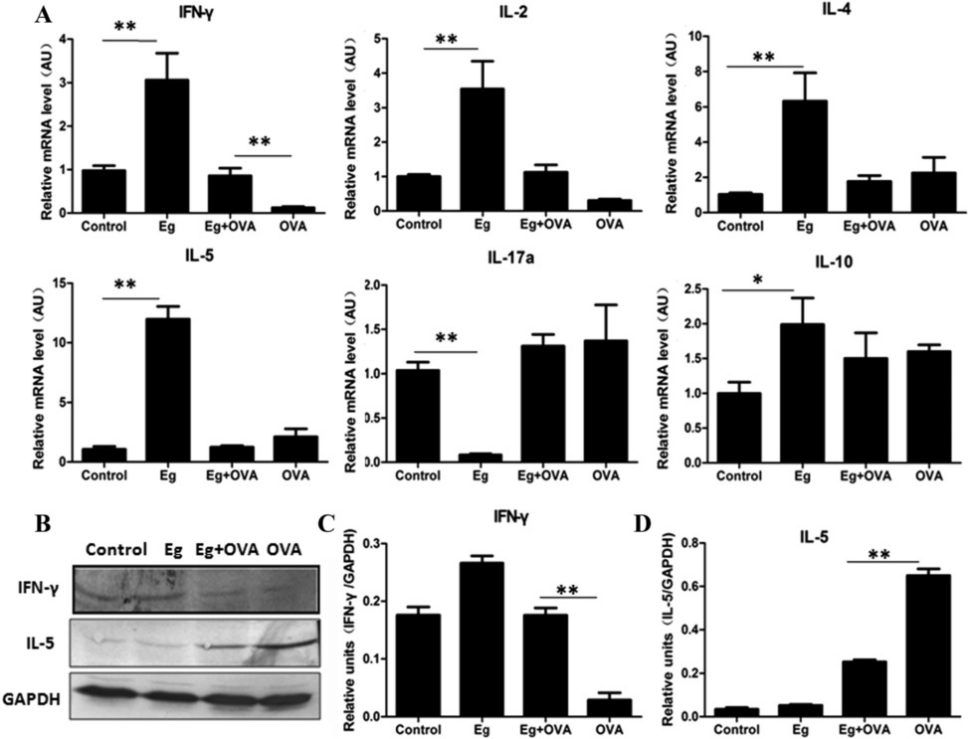
**Cytokines in the lung tissues of mice sensitized and challenged with PBS or OVA.** Cytokine mRNA expression levels were measured in the lungs by real time PCR. AU: arbitrary units **(A)**. Representative example of critical cytokines (IFN-γ and IL-5) measured by western blotting (WB) **(B)** and densitometry of WB for expression of IFN-γ and IL-5**(C,D)**. *, *p* <0 .05; **, *p* <0.01; ***, *p* <0.001. n =8 mice/group, in two separate experiments.

*E. granulosus* infection induced remarkably increased the transcriptional levels of IFN-γ, IL-2, IL-4, IL-5 and IL-10 in the lungs (Figure 4A). However, for these infected mice sensitized and challenged with OVA, only IFN-γ was higher in the lung tissues than this of OVA only sensitized and challenged mice (*p* <0.01) (Figure 4A); the transcriptional levels of IL-4 and IL-5 in Eg + OVA mice were not statistically different from the OVA mice. We then quantified the protein expression of the critical cytokines (IFN-γ, IL-5) by Western blotting. IFN-γ was higher in the lung tissue of Eg + OVA mice than that of the OVA only group (~6.03-fold). IL-5 was also significantly induced in the lungs of OVA mice. However, Eg + OVA significantly down-regulated IL-5 expression in the lung tissues compared with that of the OVA only mice (Figure 4B, C, D) (*p* <0.01).

### Effect of *E. granulosus* infection on Treg and Th17 cells in the OVA-induced mice

**Figure 5:**
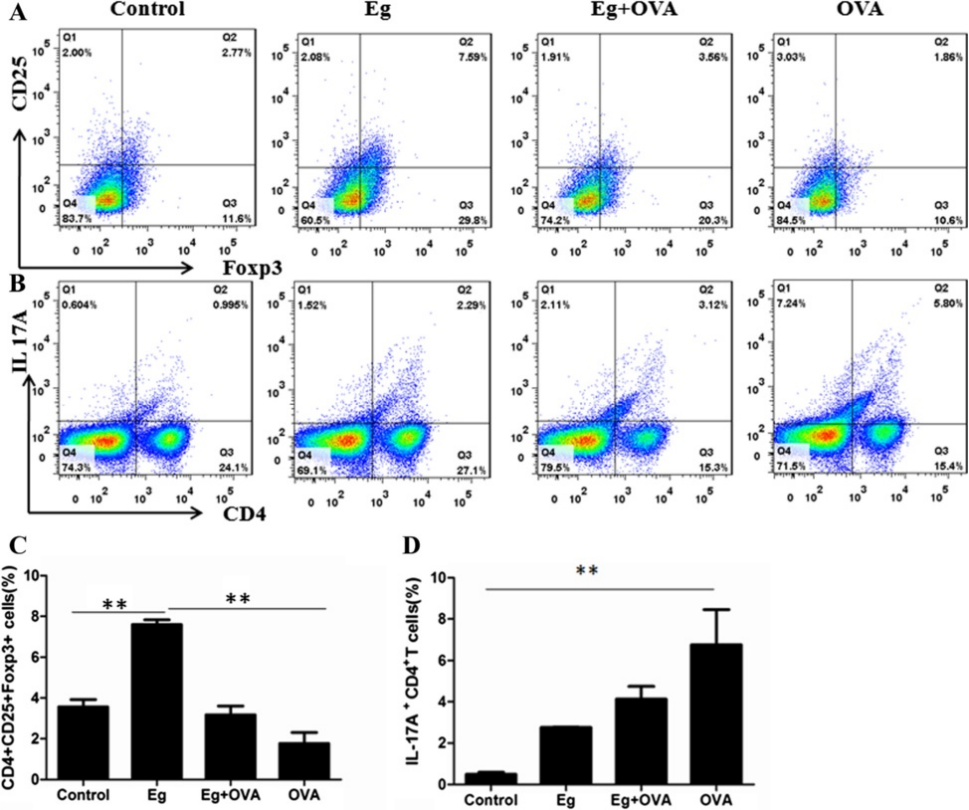
**Effects of** ***E. granulosus*** **infection on the proportion of Treg cells and Th17 cells in the spleen.** Original dot plots from FACS analysis of CD4+CD25+Foxp3+ Treg cells **(A)** and IL-17A^+^ CD4^+^ T cells **(B)** isolated from spleens. The numbers in the dot plots indicate the percent of cells within each quadrant. **C** and **D** show the percentage of CD4^+^CD25^+^Foxp3^+^ Treg cells and IL-17A^+^ CD4^+^ T cells of the splenocytes. Data are mean ± SEM of five mice, in two separate experiments. *, *p* <0.05; **, *p* <0.01.

As shown earlier, *E. granulosus* infection reduced airway inflammation; to determine whether Treg or Th17 cells were involved in this phenomenon, we further evaluated the Treg cell and Th17 cell subsets in the spleens of mice after OVA treatment compared with those of animals without *E. granulosus* infection. The population of CD4^+^CD25^+^Foxp3^+^ Tregs was not significantly changed upon OVA sensitization compared with the control group. However, the population of CD4^+^CD25^+^Foxp3^+^ Tregs increased significantly in the *E. granulosus* infected mice compared with those of the OVA mice (Figure 5A, C). Additionally, the subset of Th17 cells in the OVA-induced spleen was significantly higher in number than that of the control group (Figure 5B, D).

### Antibody responses

**Figure 6:**
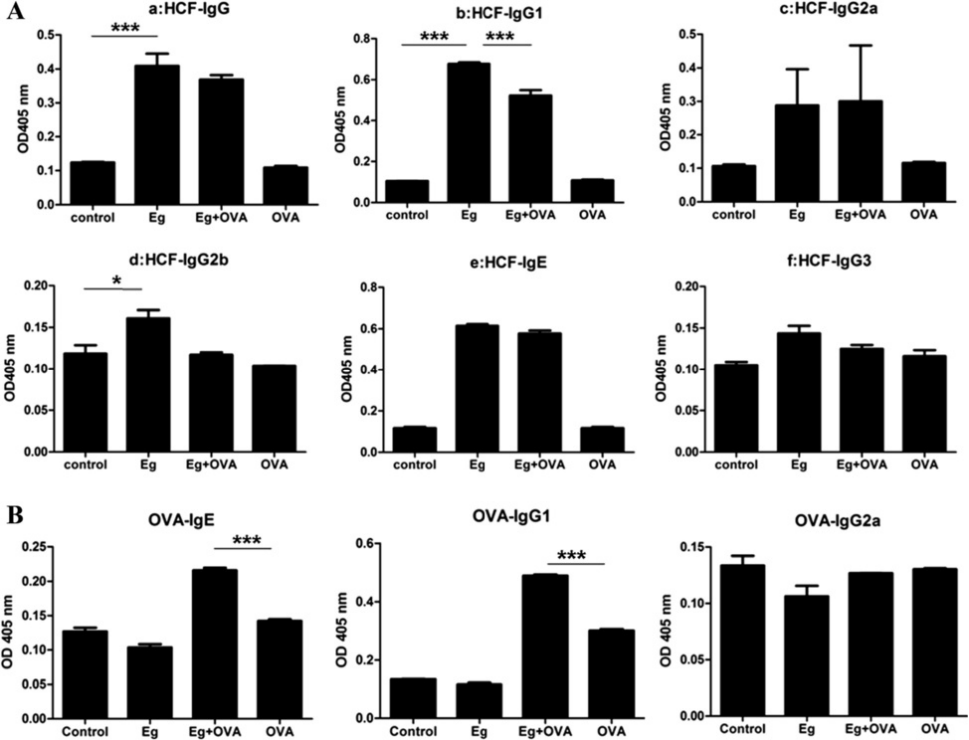
**Effects of** ***E. granulosus*** **infection on serum Ig production.** HCF-specific serum Ig levels before and after OVA challenge **(A)**. OVA-specific serum Ig levels **(B)**. *, *p* <0 .05; **, *p* <0.01; ***, *p* <0.001. n =5 mice/group, in two separate experiments.

Mice infected with *E. granulosus* had significant levels of Th2 type immunoglobulins (IgG1 and IgE) against HCF antigens in their sera compared with controls (*p* <0.001). IgG2b was also increased (*p* <0.05) in response to *E. granulosus* infection albeit at low levels. IgG3 levels remained low in all mice (Figure 6A).

After the mice were sensitized and challenged with OVA, the level of anti-OVA IgE was higher in infected than uninfected mice (Figure 6B). However, the OVA sensitization and challenge suppressed the production of HCF-specific IgG1 and IgG2b antibodies compared with their levels in *E. granulosus* infected mice (*p* <0.05) (Figure 6B). IgG2a and IgE levels against HCF antigens remained low.

## Discussion

Asthma is a chronic inflammatory disorder characterized by reversible airway obstruction, bronchial hyper-responsiveness, mucus production and eosinophilic airway inflammation [28]. A skewing of the Th1/Th2 balance towards Th2 is thought to play an important role in the development of allergic asthma [14]. It has been shown that *E. granulosus* infection regulates the host immune response, biasing a Th1/Th2 response towards a chronic Th2 response [15], which effectively minimizes host immune attack and ensures the parasite’s survival in the host for many years [29]. Given that a Th2 response is a key element for causing asthma, *E. granulosus* infection could be considered likely to enhance an asthmatic response. By contrast, however, the results of this current study reveal that an *E. granulosus* infection reduced OVA-induced pulmonary inflammation, airway mucus production, and the number of accumulated eosinophils in BALF and AHR in sensitized mice.

We infected mice by transplantation of small *E. granulosus* cysts into the peritoneal cavity. We initially infected Chinese Kunming (CKM) mice-an outbred mouse strain. The infected animals remarkably decreased asthma pathology responses (not shown). We then used BALB/c mice to repeat these experiments given that the BALB/c mouse is a recognized experimental inbred strain for allergic asthma study [30]. The infection significantly generated a predominant Th2 systemic immune response, with elevated IgG1 and IgE antibodies generated. This is similar to the response observed in human CE infection [31-36]. Histological sections of lung tissue from the sensitized and challenged with OVA showed that this allergen stimulated a large number of infiltrating eosinophils in the peribronchial regions of the lung (Figure 2A). However, mice pre-infected with *E. granulosus* significantly suppressed OVA-induced allergic airway inflammation, accompanied with peribronchial accumulation of eosinophils. In addition, the numbers of neutrophils, macrophages, lymphocytesin and eosinophils in BALF, PECs and blood showed that an *E. granulosus* infection inhibited OVA-induced eosinophil recruitment in the BALF. Detection of EMBP provides a means of determining the participation of eosinophils in the pathogenic process. We found that *E. granulosus* infection reduced EMBP expression in the lung tissue of OVA challenged mice. We also found that IL-5, which plays a critical role in the differentiation, infiltration, and activation of pulmonary eosinophils [37], was down-regulated by *E. granulosus* infection.These findings demonstrate that an *E. granulosus* infection has the potential to counteract allergic asthma-associated eosinophilic airway inflammatory responses.

As previously reported, we found that inoculation of mice with *E. granulosus* significantly increased IgE serum levels, which is a typical pro-allergic response [38]. It has been suggested (the IgE blocking hypothesis) that elevated polyclonal IgE saturates the Fcε R (IgE receptor), which may prevent the binding of antigen-specific IgE to mast and other cells central to atopic inflammation, resulting in reduced atopic symptoms [17]. Unexpectedly, we found a significant induction of OVA-specific IgE among the mice infected with *E. granulosus* suggesting that induction of polyclonal IgE is not likely to be an important mechanism of *E. granulosus* -induced protection in this model; furthermore, recent clinical studies have cast doubt on the IgE blocking hypothesis [39-41].

In addition to the increased IgE levels in the Eg + OVA group, subclass isotyping indicated that IgG1 antibodies dominated the IgG response. The observation of an enhanced IgG1 response was in concordance with the cytokine expression in serum and in supernatants of cultured splenocytes, showing a predominance of IL-4, indicating a polarized Th2 response is stimulated by *E. granulosus* infection. OVA normally induces a predominant Th2 allergic response, so the combination would likely increase the Th2 response. However, in contrast, our results showed that the combination of *E. granulosus* infection and OVA sensitization and challenge down-regulated the Th2 response in the lungs. In addition, we identified that *E. granulosus* infection induced a significant IFN-γ production in their lung tissues. However, the combination of *E. granulosus* infection and OVA inhibited the expression of this cytokine and others including IL-2, IL-4, IL-5 in the lungs compared to the *E. granulosus* infected mice.

Several studies have demonstrated that IL-17 is up-regulated in lung tissues, BAL fluids, sputum, and peripheral blood of patients with allergic asthma [42-48]. In mouse models of asthma, Th17 cells enhance Th2 cell-mediated eosinophilic airway inflammation [49] and it has been shown that IL-17 depletion in OVA-sensitized mice attenuates the inflammatory response [44,50]. These observations have indicated that IL-17 participates in the development of allergic asthma. As research on the role of IL-17 in allergic airway inflammation is still at an early stage, there is limited literature addressing the regulation of IL-17 and its related molecules in asthma. IL-17A is a key pro-inflammatory cytokine in the T helper 17 pathway [51] and it plays a critical role in host defense and inflammation. Some animal studies revealed that helminthic infections ameliorate the severity of autoimmune diseases by reducing the Th17 response. In the current study, we found that IL-17A levels were significantly increased in OVA induced mice both in splenocyte culture and serum. However, compared with OVA Group, the mice in Eg + OVA Group showed a low concentration of IL-17A in serum, implying that *E. granulosus* infection may down-regulate Th17 expression.

In contrast, IL-10 was significantly higher in the Eg + OVA group than in the OVA induced group after measurement of its concentration in splenocyte culture supernatants. IL-10 is a regulatory cytokine that performs crucial functions in parasitic infections. Patients infected with CE have a slight decrease in peripheral IL-17 and an increase in peripheral IL-10 levels compared with healthy controls [52]. Many previous reports have demonstrated that the regulation of asthma reactions by parasitic infection is related closely to the up-regulation of IL-10 [53-55]. IL-10 has been suggested as a treatment of asthma because of its immunosuppressive and anti-inflammatory properties. Recent studies have shown that the IL-10 gene can significantly reduce the expression of IL-17 [56,57]. We thus speculate that *E. granulosus* infection may decrease the severity of allergic asthma by enhancing IL-10 expression and down-regulating the expression of IL-17A.

## Conclusions

We demonstrate here that a prior infection with *E. granulosus* can effectively suppress allergic airway eosinophilic inflammation in an OVA-induced murine allergic asthma model. Furthermore, we provide experimental evidence that an *E. granulosus* infection enhances IL-10 expression and down-regulates the production of IL-17A and IL-5, which in turn reduces the severity of OVA-induced airway inflammation. The molecules produced by *E. granulosus* which down-regulate the Th17 and IL-5 responses may prove useful agonists for prevention and treatment of allergic asthma.

## Additional file

Additional file 1: Table S1. Primers for quantitative real time PCR to detect cytokines of mice.
