# Supplementary material for: Echinococcus granulosus infection reduces airway inflammation of mice likely through enhancing IL-10 and down-regulation of IL-5 and IL-17A
Source: Parasit Vectors. 2014 Nov 20;7:522. doi: 10.1186/s13071-014-0522-6 (PMC4256745; doi:10.1186/s13071-014-0522-6)
Supplement: Additional file 1: Table S1. — Primers for quantitative real time PCR to detect cytokines of mice. [file 13071_2014_522_MOESM1_ESM.doc]

**Table S1 Primers for quantitative real time PCR to detect cytokines of mice.**

| **Gene** | **Genbank**  **Accession** | **Primer Sequences** | **Expected**  **Size** | |
| --- | --- | --- | --- | --- |
| IFN-γ | NM_008337 | F:5’-ATGGCTATTTCTGGCTGTTACT-3’  R:5’-AATGACGCTTATGTTGTTGCTG-3’ | | 239 bp |
| IL-2 | NM_008366 | F:5’-GGCATGTTCTGGATTTGACTC-3’  R:5’-TCCATCTCCTCAGAAAGTCCA-3’ | | 166 bp |
| IL-4 | NM_021283 | F:5’-GGTCTCAACCCCCAGCTAGT-3’  R:5’-GCCGATGATCTCTCTCAAGTGAT-3’ | | 102 bp |
| IL-5 | NM_010558 | F:5’-TCAGGGGCTAGACATACTGAAG-3’  R:5’-CCAAGGAACTCTTGCAGGTAAT-3’ | | 168 bp |
| IL10 | NM_010548 | F:5’-CTTACTGACTGGCATGAGGATCA-3’  R:5’-GCAGCTCTAGGAGCATGTGG-3’ | | 101 bp |
| IL-17A | NM_010552 | F:5’-TATCCCTCTGTGATCTGGGAAG-3’  R:5’-ATCTTCTCGACCCTGAAAGTGA-3’ | | 160 bp |
| Muc5ac | NM_010844 | F:5’-AAAGACACCAGTAGTCACTCAGCAA-3’  R:5’-CTGGGAAGTCAGTGTCAAACCA-3’ | | 139 bp |
| GAPDH | NM_008084 | F:5’-AGGTCGGTGTGAACGGATTTG-3’  R:5’-TGTAGACCATGTAGTTGAGGTCA-’ | | 123 bp |
